# Paralysis of Liver

**Published:** 1878-01

**Authors:** P. H. Hayes


### PARALYSIS OF THE LIVER.

P. H. HAYES, M. D.

A farmer came into my office yesterday, saying he has gradually and for some months been losing strength, and that now he can't even do his "chores" night and morning. He suffers very much from the cold. He is thin, his features have a pinched and shrunken aspect, and his skin is of a dingy, faint lemon color. On enquiring into his history, I find he is one of the class of farmers that drives business, and keeps hired men at work by setting them an example, and that he has been in the habit, when his own strength flagged, of "keeping up" on "Perry Davis's Pain Killer," taken, secundem directionem, into the stomach, and this has been his habit for years. Last year, for a change, he put in cider and run for six months on that, and thinks he did well. Quite recently he returned to his old and tried friend, the "Pain Killer," but sad to say, instead of bringing him up again to working order, he says "*it went back on him*," and this is what really sent him to me. On careful examination, I find the liver is scarcely two-thirds its natural size, the digestive organs flat and flabby, and he tells me the stools are very light-colored.

This patient has, by overwork and stimulation, brought on a paralysis of the liver, and a low type of chronic inflammation in the organ, and these in turn have brought on a shrinking of the liver by the waste of those parts which secrete the bile. You will observe that I put paralysis first and chronic inflammation next, for there is no doubt that paralysis of the extremely minute vessels, which enter into the bile-making system of the liver, is the first in natural order, and that chronic inflammation and shrinkage are secondary and necessary consequences.

It must not be forgotten that rapid absorption takes place by the veins of the stomach, and that all these veins send their blood, by the route of the portal system, directly to the liver, and that, therefore, everything absorbed directly from the stomach, must first pass through the liver before it gets into the general circulution.

Now, how did my patient bring on paralysis, chronic inflammation and contraction of the liver? The *Pain Killer* he says is composed of stimulating gums, red pepper and other hot things, "cut by alcohol." The alcohol is doubtless the chief black sheep in this compound, but rendered doubly powerful for evil by the other hot things which it so rapidly conveys to the liver. Here the first effect is to stimulate to an unusual flow of bile, and this stimulates the digestion of fats and oils, sugars and starch, which undergo conversion into blood principally in the bowels and below the point where the bile enters. The bowels were rendered more active, more food is actually appropriated, and the man finds that he can do a "power of work" when under the influence of the "*Killer*," not only for the reason I have mentioned, but because the hot stimulants are also in the blood, and bringing, for the time being, all the powers and actions of life into vigorous action. But after awhile my patient finds when he stops taking the "*Killer*" that his bowels won't act; that they become blocked; that his food sets heavy on his stomach; that he loses flesh and strength, and that now he can no longer set his hired man an example.

But as a stomach and liver weakened by stimulants cry out for more and stronger, so he turns to cider ("after it is a little old"), and makes up by quantity what it lacks in fire, and goes on for nearly another year. And now he stops cider and turns once more to his old friend, the "*Killer*," and grief of griefs, and this old friend, as if finally to vindicate its title, "goes back on him;" he can get no comfort or stimulus out of it; the long irritated stomach and liver are worn out by it, and now "not able to lift his hand to do a single chore," and with "weak spells" by day and troubled dreams by night, he turns to his physician for help.

There is another form of paralysis of the liver which I am tempted to notice right here, in which the stages of stimulation collapse, paralysis and inflammation are developed in a few weeks, and sometimes even in a few days. A very intelligent missionary lady told me that when she first went to Africa the malaria was so poisonous to her that for some weeks the climate was exhilirating almost to intoxication; that then came the dreadful chills, with intense fever and inflammation of the liver, demanding, as she said, for the chills, a hundred and fifty grains of quinine in a day, and for the liver, a score of leeches at a single sitting. Now, during the progress of this woman's acclimation she went through with this exhiliration by the malarious poison, paralysis and inflammation of the liver, chills and slow recovery, many times, each time the symptoms less violent, yet demanding more and more powerful dosing, until finally, the liver being nearly completely paralyzed, she returned to America a wreck and remnant of her former self.

What are called "*Billious Attacks,*" are no doubt partial and temporary paralyses of the stomach. Indeed, I believe the two things occur so often together, and from the same causes, that it is hardly worth while to try to separate them. The liver and stomach are supplied by nerves from the same roots and centers, and what the stomach absorbs is in an instant more to be found in the liver. These partial and temporary paralyses of the liver are extremely common, and are induced by high living and inactive habits of life. They are also induced by high living and excessive labor of body or mind. They are also induced by excess in tea or coffee. When we take a very common breakfast of hot cakes, hot bread, melted butter, and strong coffee, we find in this the elements of billous disorder from an overtaxed liver; and when at noon the dinner consists of pork, in its own fat, hot sauces and condiments, followed by pastry for dessert, no liver will bear such overtaxing, except it be that of a man who labors hard and continuously in the open air. It is easy to see that if wine or cider be added to the dinner, or the inevitable green tea, or coffee, the case is still stronger against the liver. If we go no further than already indicated in our search into dietetic causes, we have already a sufficient explanation of the actual fact that almost every one is in a sense and in a degree "*bilious.*" This is one of the grand reasons why we so rarely see a truly fine and clear complexion. The skin is tinged with bile, and unhealthy or vitiated bile at that. But of all dietetic habits, the surest to paralyze the liver is eating late and hearty suppers. The body runs down in vigor as night approaches and darkness deepens, and far on toward morning it continues to descend to a lower condition of circulation, warmth and nerve power. To eat a hearty meal and late at night is to impose a heavy task upon the liver just as its time for absolute rest had come. That such persons should have bilious headaches, fetid breath, bilious vomiting, and a vile, bilious taste in the mouth in the morning, should not be considered surprising. I ought to say that the type of liver paralysis caused by high living and inactive habits of life is not usually complicated by chronic inflammation, contraction and hardening of the organ, but by a condition nearly directly opposite. The liver becomes enlarged, sometimes to twice its natural size, it is pale, flabby, and filled with fatty matter. For ages in different parts of Southern Europe there has been served upon the tables of the rich, what is esteemed one of the richest of all delicacies, pies made from the fatty livers of geese. The geese are kept in a dark place and so closely confined, usually in a basket, that they cannot stir, and here they are gorged with fatty foods until their livers become enormously enlarged; indeed, so large as to weigh more than the entire remainder of the animal. You can see the pies made from such livers advertised now in the windows of first class city restaurants, under their French title, *Pate De Fois Gras.* Pliny, the Roman historian and naturalist, who flourished about the year 70 of the Christian era, mentions these large goose livers, and says it is uncertain whether Scipio Metullus, of consular dignity, or M. Sestius, a Roman Knight, was the great discoverer of this excellent dish.

If now we go back to the case of the farmer with which we began, we shall find in it a type of the natural history of stimulation. Working in the open air, for years he suffered but little from the habit of taking, in small doses, the fiery alcoholic preparation known as Perry Davis's Pain Killer. But by-and-by he finds he cannot leave it off; his stomach will not only not digest his food without it, but will not even receive it without this stimulant or its equivalent. Another year comes around, and now his stomach and liver will not receive the stimulant at all, nor its equivalent in any form. He can only digest a little of the simplest food, and cannot lift his finger to do a chore. It is worthy of observation that this man pursued the habit of stimulation under the most favorable circumstances possible, living a regular and industrious life in the open air, and with a vigorous constitution and in the prime of life. But his case demonstrates that there is no such thing as perpetual exaltation by stimulation; that there must come a final recoil, a final paralysis, more or less complete, of the digestive and nutritive organs among which the liver has been found to rank far higher than was formerly supposed.

